# Estimation of dynamic flux profiles from metabolic time series data

**DOI:** 10.1186/1752-0509-6-84

**Published:** 2012-07-09

**Authors:** I-Chun Chou, Eberhard O Voit

**Affiliations:** 1 Integrative BioSystems Institute and The Wallace H. Coulter Department of Biomedical Engineering, Georgia Institute of Technology and Emory University, 313 Ferst Drive, Atlanta, GA, 30332, USA

**Keywords:** Biochemical systems theory, Dynamic flux estimation, Metabolic pathways, Parameter estimation, Structure identification, Time series data

## Abstract

**Background:**

Advances in modern high-throughput techniques of molecular biology have enabled top-down approaches for the estimation of parameter values in metabolic systems, based on time series data. Special among them is the recent method of dynamic flux estimation (DFE), which uses such data not only for parameter estimation but also for the identification of functional forms of the processes governing a metabolic system. DFE furthermore provides diagnostic tools for the evaluation of model validity and of the quality of a model fit beyond residual errors. Unfortunately, DFE works only when the data are more or less complete and the system contains as many independent fluxes as metabolites. These drawbacks may be ameliorated with other types of estimation and information. However, such supplementations incur their own limitations. In particular, assumptions must be made regarding the functional forms of some processes and detailed kinetic information must be available, in addition to the time series data.

**Results:**

The authors propose here a systematic approach that supplements DFE and overcomes some of its shortcomings. Like DFE, the approach is model-free and requires only minimal assumptions. If sufficient time series data are available, the approach allows the determination of a subset of fluxes that enables the subsequent applicability of DFE to the rest of the flux system. The authors demonstrate the procedure with three artificial pathway systems exhibiting distinct characteristics and with actual data of the trehalose pathway in *Saccharomyces cerevisiae*.

**Conclusions:**

The results demonstrate that the proposed method successfully complements DFE under various situations and without *a priori* assumptions regarding the model representation. The proposed method also permits an examination of whether at all, to what degree, or within what range the available time series data can be validly represented in a particular functional format of a flux within a pathway system. Based on these results, further experiments may be designed to generate data points that genuinely add new information to the structure identification and parameter estimation tasks at hand.

## Background

A grand challenge of biomathematical modeling is the conversion of a biological system into a computational structure that formalizes the underlying system. An important and very challenging component of this process is the estimation of parameter values. The task is typically pursued with one of two generic approaches, namely a forward (bottom-up) or an inverse (top-down) method. Until recently, essentially all models of metabolic pathway systems were developed according to the first strategy, that is, by characterizing model components and processes one at a time and subsequently merging all “local” information about kinetic reaction steps into one comprehensive dynamic model. Although this forward approach is theoretically straightforward, implementation procedures often fail and, moreover, have intrinsic disadvantages [1]. For instance, the necessary information is usually obtained *in vitro* and from different experiments, so that there is no guarantee that it is entirely compatible and consistent.

The second, top-down approach uses data that characterize the entire system and attempts to estimate all parameter values at once with a sophisticated optimization algorithm. Specifically, this type of method employs time series data that describe the full dynamic response of a pathway system to some stimulus, such as an environmental stress (*e.g.*, heat shock) or the availability of food (*e.g.*, glucose uptake). In contrast to the local data obtained from traditional experiments, the great appeal of using these types of “global” data is that most, if not all, measurements are taken from the same biological system under the same conditions. Furthermore, these data contain enormous and essentially unadulterated information about the structure, dynamics and regulatory mechanisms that govern the biological system under investigation. The main drawback of top-down approaches is that the actual extraction and integration of this information into fully functional, explanatory models is challenging. In fact, more than one hundred articles addressing this task appeared within the past ten years. They focused on various aspects of the estimation process, but most of them were dedicated to the main issue of optimizing parameter values against the observed time series data (*e.g.*, [2,3]).

Whether a forward or inverse approach is used, the estimation of parameter values necessitates assumptions regarding the functions or rate laws that describe the reactions of interest. As a prominent example, the typical default for enzymatic reactions in a metabolic pathway is the Michaelis-Menten rate law (MMRL) or one of its variations. While such assumptions are understandable, they create an immediate conundrum. Namely, the true mechanisms governing a biological process are in reality unknown or at least unclear. As a result, the estimation process is from the start unguided, uncertain, or maybe even based on modestly or entirely wrong assumptions. Also, descriptions of more complex enzyme mechanisms contain numerous parameters if several substrates or reactions are involved, so that the alleged functions cannot be identified from the typically sparse data [4,5].

In addition to the troublesome issue of model selection, most proposed methods for estimation from time series data face significant problems related to the data themselves, to inefficient algorithms, and to a variety of computational issues. To complicate matters further, these issues are usually superimposed. The data may be overly noisy, incomplete, collinear with each other, or non-informative. The computational algorithms are often slow to converge, converge to a locally but not globally optimal solution, or do not converge at all. Finally, there is a mathematical issue, especially for systems with many parameters, namely that a system may admit solutions that are distinctly different yet equivalent, or essentially equivalent, with respect to the residual error. This type of result, referred to as sloppiness and unidentifiability, may be due to redundancies in candidate parameter sets and has received much attention in recent times [6-8].

A different type of sloppiness may be caused by the fact that different model structures may give essentially identical residual errors. For instance, several probability density functions often model the same data equally well [9]. Moreover, two “wrong” structures or representations within a model may permit compensation of errors between different terms or equations [10]. It is not even clear whether the residual error (SSE) is always the best metric for the quality of fit [11]. For instance, the “best” models in terms of having the smallest SSE tend to have too many parameters and therefore encounter over-fitting problems. This issue can be serious, because an over-fitted model often lacks the capacity of extrapolation and predictive power with respect to data not used in the estimation or untested conditions. Therefore, it is necessary to develop tools for the evaluation of model validity and quality beyond residual errors. For instance, one should establish criteria to determine the appropriateness of the chosen mathematical representations, develop methods for assessing whether residual errors are due to idiosyncrasies or noise in the data, and develop diagnostic tools of discriminating between valid and invalid model structures.

Recently we proposed a novel approach to metabolic systems estimation, called *Dynamic Flux Estimation* (DFE), that ameliorates several of the issues listed above [10]. DFE is executed in two distinct phases. The first phase consists of an entirely model-free data analysis that requires minimal assumptions and reveals inconsistencies within the data, and between data and the alleged system topology. Generally, the system is represented as a set of ordinary differential equations (ODEs) so that the instant change in each metabolite (*i.e.*, its derivative) equals the sum of fluxes that enter or leave the metabolite pool:

(none1) $$\frac{dX_{i}}{dt}={\dot{X}}_{i}=\sum Influxes-\sum Effluxes\;\text{.}$$

The left-hand side of this ODE can be interpreted as the slope of the time course of the variable *X*_*i*_ at a given point in time. Therefore, assuming that the time series data are more or less complete and smooth—or can be validly smoothed (see later) —one can estimate the slope of the time course at each time point and substitute the slopes for the derivatives. If the system contains *N* equations, and if data are measured at *K* time points, this substitution decouples the system of *N* differential equations into *N* sets of *K* algebraic equations each. This system is linear in the fluxes and can be assessed with methods of linear algebra. In particular, it is easily solved at each time point if the system has full rank.

The result of this first phase of DFE is a representation of each flux as a numerically characterized function of time and as a function of all contributing metabolites. This representation is not explicit, but purely numerical and consists of points in plots of flux *vs.* time or flux *vs.* metabolites and modulators. The second phase of DFE addresses the mathematical formulation of each process in the biological system by attempting to convert these numerical plots into mathematical representations, such as a Michaelis-Menten or Hill rate law or a power-law description. In contrast to most other methods, where a functional form had to be assumed *a priori*, this step allows quantitative diagnostics of whether a candidate of a mathematical formulation may be appropriate, at least within certain ranges of the contributing variables. The subsequent determination of parameter values is now much easier, because it involves explicit functions that are addressed one flux at a time.

DFE offers substantial advantages. It makes almost no assumptions and is straightforward if the right data are available. It reveals inconsistencies within the data, avoids compensation among and within equations, and permits quantitative diagnostic tools of whether the assumed mathematical formulations are appropriate or in need of improvement. In addition, since DFE identifies parameters based on explicit single-flux representations, the estimation of parameter values is much easier and more reliable than in other top-down approaches. As a result, DFE promises significantly improved extrapolation capacity toward new data or experimental conditions.

Alas, DFE also has limitations and drawbacks. First, it requires more or less complete time series data that characterize the investigated system. These data are still relatively seldom, although they are being generated at an increasing rate and with rapidly improving quality. Second, and arguably more limiting, a unique solution of the flux equations in the first phase of DFE is only possible if the flux system is of full rank. However, most actually pathway systems contain more fluxes than metabolites and are therefore underdetermined.

Several constraint-based optimization techniques have been proposed for stoichiometric analyses of underdetermined metabolic systems [12]. They have become a mainstay of flux balance analysis (FBA [13]) and work well under steady-state and pseudo-steady-state (PSS) assumptions [14-17]. Mahadevan and co-workers [18] extended the traditional FBA to account for dynamics and presented two different formulations: the dynamic optimization approach (DOA) and the static optimization approach (SOA). DOA involves optimization over the entire time period to obtain flux profiles over time, while SOA involves dividing the batch time into several time intervals and solving the instantaneous optimization problem at the beginning of each time interval. These methods basically are variations of FBA and need, for the determination of flux profiles at each time point, constraints and objective functions, which describe some goal the cell aims to reach. For the case of microbial systems, a reasonable objective may be maximization of the growth rate. However, determining an unbiased objective function in a eukaryotic system is often difficult.

In contrast to these methods that require objective functions, we proposed extending DFE with the infusion of additional information [19]. We distinguished four cases. First, the connectivity of the systems is not fully known or some of the connections are uncertain. Second, some of the time series data were not measured, although it is known how the corresponding metabolites are involved in the pathway. Third, the system contains “missing” metabolites which are neither known nor measured, but in actuality affect the system significantly. And fourth, the flux system is underdetermined, even though the time series of all relevant metabolites are measured.

The first issue might be ameliorated by methods developed for structure identification of unknown of ill-characterized pathways. These methods include a wide spectrum of techniques, such as perturbation methods, causality models, correlation-based approaches, or probabilistic models, some of which are based on time series data (see [2] for review). The lack of certain data in the second case could be complemented by deducing the unknown time profiles from time series of neighboring metabolites, if the corresponding enzymatic information is available for fluxes producing and degrading a metabolite in the equation. However, this approach of using kinetic information obtained *in vitro*, or maybe even from different organisms, is naturally problematic due to some degree of bias and uncertainty. A possible solution strategy for the third case is to check the mass balance in the entire system throughout the time period. If significant changes in mass balance are observed, additional biological insight will be needed to check the pathway model and identify possible sources of leakage or gain of mass. If the masses are more or less balanced, it is still possible that important fluxes or metabolites are missing. However, there is currently no obvious defense in this situation. Finally, to complement an underdetermined flux system, some of the fluxes need to be estimated with information from other sources. For instance, it might be possible to obtain fluxes directly from experiments, but such data are rare. As an alternative, one may assume the functional form for an enzymatic reaction, and if corresponding kinetic information is available, for instance from BRENDA [20], parameter values may be estimated for this functional form. As a variation on this strategy, one could assume some canonical model, such as power-law functions [21] or lin-log approximations [22,23], if some of the variables and fluxes operate within relatively small ranges. Clearly, this option runs counter to the model-free nature of DFE, but might be the only feasible solution. Instead of using kinetic information, one could also select some of the decoupled equations and use optimization methods to fit the selected model to the time series data (*e.g.*[24,25]).

Although we presented proof of concept that the different approaches described above can be used to supplement DFE, these approaches are not always optimal, because they require additional information and assumptions that are *a priori* not validated. The question thus arises: can we directly squeeze additional information out of the time series data, without the need of further assumptions and additional information? And if so, under what conditions is that possible? Providing at least partial answers to these questions is the topic of this article.

Specifically, we propose here a distinct approach to supplementing DFE with information hidden in suitable metabolic time series. Extracting this information permits the determination of a sufficient subset of fluxes to execute DFE on the rest of the flux system. In contrast to all other solutions presented so far for the complementation of DFE, the method proposed here does not require any assumptions regarding the mathematical representation of the fluxes. Furthermore, kinetic information or knowledge of the functional forms of the enzymatic reactions is not required. We will demonstrate in the following that the proposed method can succeed even if some of the time series data are not measured or when there is mass leakage in the pathway systems. In addition, the new method allows us to address a recurring unanswered question, namely how many time series data are needed to estimate the structure and parameters of a system.

Specific details of the proposed approach are presented in the *Methods* section. While the methodological details require some technical discussion, the concept of the proposed method may be best explained with the following simple example. Suppose a metabolic system contains the equation ${\dot{X}}_{i}=v_{i}^{+}(X_{j})-v_{i}^{-}(X_{i})$, which is typical for a reaction between *X*_*j*_ and *X*_*i*_, combined with the degradation of *X*_*i*_ within a linear section of a pathway system:

(none2) $$X_{j}\to X_{i}\to$$

Suppose we have time series data, so that we can estimate ${\dot{X}}_{i}$ for every measured time point with sufficient accuracy. Suppose further that the time series data are such that we have *m* time points (in the same or in different datasets) where *X*_*i*_ has the same value (*e.g.*, *c*_i_), whereas *X*_*j*_ has a different value at each of these time points. It is reasonable to assume that $v_{i}^{-}$ is a function in a strict mathematical sense, which means that $v_{i}^{-}(c_{i})$ has one unique (although yet unknown) value *vc*_*i*_. If so, we have *m* equations of the type ${\dot{X}}_{i}=v_{i}^{+}(X_{j})-vc_{i}$, where the values of *X*_*j*_ and ${\dot{X}}_{i}$ are known directly from the data and *vc*_*i*_ always has the same value. Using these quantities, the methods proposed here allow us to estimate the functional format of $v_{i}^{+}(X_{j})$, at least over some pertinent range of *X*_*j*_ values. Once we know $v_{i}^{+}(X_{j})$, we can determine $v_{i}^{-}(X_{i})$. Thus, we now have numerically quantified two fluxes, which reduce the discrepancy between the number of equations and the number of independent fluxes. Repeated application of the method allows DFE for the entire system. An illustrative $v_{i}^{+}$ example is shown in *Methods* and other examples are presented in the *Results*. If the function depends on more than one variable, the procedure is the same in concept but more complicated in detail (see Additional file 1).

## Methods

The proposed method offers a systematic strategy to extend DFE and to ameliorate its limitations. Just like DFE, the proposed method starts with an optional data preprocessing step, but without any assumption regarding the functional formats of the fluxes in the system. First, the experimental data are tested for mass conservation to make sure no mass is lost or gained during the observed time period. If the data do indicate losses or gains in mass, it is useful to locate possible branches off the main pathway(s) and to account for the changes in total mass of the metabolites in the pathway [19]. Second, the time series data are smoothed as necessary, which makes it easier to estimate the slopes of all time courses at a given number of time points, using different numerical techniques. These established smoothing methods include splines, artificial neural networks, as well as different types of filters, such as the popular Kalman, Savitzky-Golay, Whittaker, or Eilers filter (see [2,26,27] for applicable methods). In parallel to these data preprocessing steps, the pathway structure (the system topology) is used to generate a system of symbolic equations describing the dynamics of the system. The generic format for such a representation may be written as

(1) $${\dot{X}}_{i}=\sum V_{\mathrm{ij}}^{+}(X_{1},\ldots ,X_{n})-\sum V_{\mathrm{ij}}^{-}(X_{1},\ldots ,X_{n})\text{,}\;i=1,\ldots ,n\text{,}$$

where *X*_*i*_ denotes the concentration or amount of a variable or variable pool and *n* is the total number of time-dependent variables in the system. The functions $V_{i}^{+}$ and $V_{i}^{-}$ represent reactions or fluxes entering or leaving the quantity *X*_*i*_, respectively. Substituting slope estimates for the differentials in this system of equations decouples the ordinary differential equations (ODEs) and results in a system of fluxes that is linear at each time point *t*[21,28,29]. The algebraic equations may be represented in matrix format as

(2) $$s(t_{j})=N\times v(t_{j}),\;j=1,\ldots ,K\text{,}$$

where **s** is a vector of slopes, **N** is the stoichiometric matrix, **v** is a vector of fluxes, and *K* is the number of time points *t*_1_*, t*_2_*,…, t*_*j*_*,…, t*_*K*_ where measurements are available.

Next we check the rank of the linear set of algebraic equations in Eq. (2). The system can be easily solved at each time step to obtain dynamic profiles of all fluxes if the system has full rank. Over-determined systems may be solved by pooling fluxes, the use of pseudo-inverse methods, or regression. However, if the system is underdetermined, the solution space is infinite. To overcome this issue, some of the fluxes need to be estimated independently, until the system has full rank and can be solved uniquely. Elsewhere we showed that additional information maybe used to characterize selected fluxes [19]. Here, the goal is to estimate some fluxes directly from the time series data, without evoking other sources of information.

**Figure 1:**
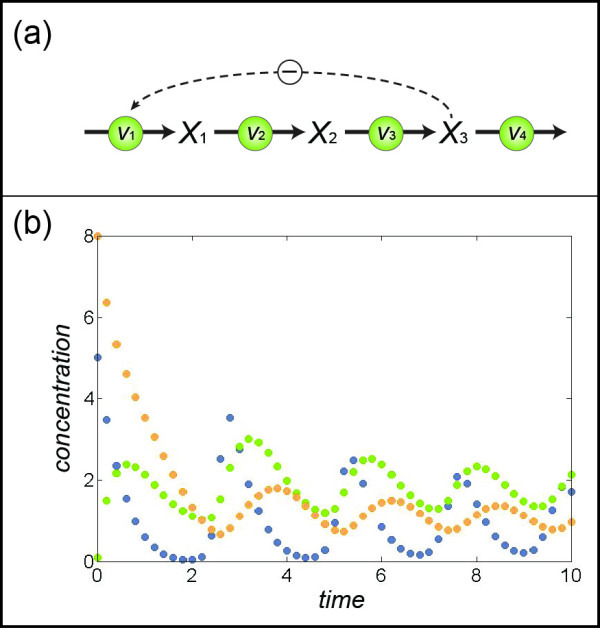
**(a) Generic three-variable linear pathway with feedback inhibition (Eqs. (3**–**4)).(b)** Time series data, consisting of 50 artificial “measurements” that were generated with initial conditions *X*_1_(*t*_0_) = 5, *X*_2_(*t*_0_) = 0.1, and *X*_3_(*t*_0_) = 8; *X*_1_, *X*_2_, *X*_3_ are represented by blue, green, and orange dots, respectively.

As an introductory example, consider a linear part of a pathway with feedback inhibition as shown in Figure 1(a). The equations that describe the system in terms of fluxes are

(3) $$\begin{array}{l} {\dot{X}}_{1}=v_{1}-v_{2}\\ {\dot{X}}_{2}=v_{2}-v_{3}.\\ {\dot{X}}_{3}=v_{3}-v_{4} \end{array}$$

The system could be part of a larger pathway system, but for this illustration the context is not relevant. For the illustration, fluxes were generated with a mix of power-law and Hill functions, namely

(4) $$\begin{array}{l} v_{1}=1.5\;X_{3}^{-6}\\ v_{2}=2.4\;X_{1}^{0.8}\\ v_{3}=\frac{V_{\max}X_{2}^{3}}{K_{M}^{3}+X_{2}^{3}},\\ v_{4}=2\;X_{3}^{0.75} \end{array}$$

where *V*_*max*_ = 5 and *K*_*M*_ = 2. We use these settings to create artificial data, but subsequently assume no knowledge of the functions or parameters in Eq. (4).

Suppose time series data were measured and they are without noise (Figure 1(b)). Eq. (3) immediately indicates that the flux system is underdetermined and therefore has infinitely many solutions. A unique solution could be obtained if one of the fluxes could be determined independently. To achieve this independent determination, one may choose any one of the three equations in the system. For ease of computation, one will typically prefer an equation that contains as few fluxes and as few substrates and modulators as possible. In this linear system, all equations have two fluxes and each of them depends on only one metabolite, so that there is no advantage to choosing one equation rather than another.

Generically, we intend to solve the fluxes in the *i*^th^ equation, which here happens to have only two fluxes, namely one influx (*v*_*in*_) going into the pool *X*_*i*_, and one efflux (*v*_*out*_) leaving this pool. The flux *v*_*in*_ depends only on the precursor *X*_*in*_ of *X*_*i*_ and *v*_*out*_ depends only on *X*_*i*_ itself; to minimize confusion, we call this variable generically *X*_*out*_. Extracting the *i*^th^ equation from Eq. (1), we thus obtain, in general terms,

(5) $${\dot{X}}_{i}=v_{\mathrm{in}}-v_{\mathrm{out}}\text{.}$$

The functional form of neither flux is assumed to be known. Substitution of derivatives with slopes results in *K* equations of the type

(6) $$S_{i}(t_{j})=v_{\mathrm{in}}(t_{j})-v_{\mathrm{out}}(t_{j})\;,\;j=1,\ldots ,K\text{.}$$

As a specific illustration, consider the second equation $\left({\dot{X}}_{2}=v_{2}-v_{3}\right)$ in Eq. (3), where *v*_2_ depends only on the precursor *X*_1_ and *v*_3_ depends only on *X*_2_. We substitute the derivative ${\dot{X}}_{2}$ with slopes that can be measured directly from the time series data, possibly upon smoothing. For a total of 50 time points, one thus obtains 50 algebraic equations of the type

(7) $${\dot{X}}_{2}\approx S_{2}(t_{j})=v_{2}(t_{j})-v_{3}(t_{j})\;,\;j=1,\ldots ,50.$$

It is reasonable to assume that the in- and effluxes are true functions in a mathematical sense. Thus, since *v*_*in*_ depends only on *X*_*in*_, *v*_*in*_ must have one and only one value for every given value of *X*_*in*_. In particular, if *X*_*in*_ assumes the same value at two different time points, *v*_*in*_ must have the same (so far unknown) value at both time points as well. In the illustration example, *v*_2_ depends only on *X*_1_. Thus, for every value of *X*_1_ there is one and only one value of *v*_2_. The proposed method therefore requires a screening of the available datasets with the goal of identifying different situations where *X*_*in*_ has some fixed value *X*_*in_const*_. For all these situations, *v*_*in*_ also has some fixed value *v*_*in_const*_. Since we do not know the functional form of *v*_*in*_, we cannot directly compute this value *v*_*in_const*_. However, we do know that this value is very similar for all situations where *X*_*in*_ ≈ *X*_*in_const*_. Thus, for the set of all *X*_*in*_ ≈ *X*_*in_const*_, Eq. (6) has the form

(8) $$S_{i}(t_{j})=v_{in\_const}(t_{j})-v_{\mathrm{out}}(t_{j})\;\text{.}$$

In the illustrative example, we screen the available data sets and search for different situations where *X*_1_ has the same fixed value *X*_1*c*_ and, thus, *v*_2_ also has the same (yet unknown) value *v*_2*c*_. Thus, for the entire set of all *X*_1_ ≈ *X*_1*c*_ the second system equation has the form

(9) $$S_{2}(t_{j})=v_{2c}(t_{j})-v_{3}(t_{j})\text{.}$$

**Figure 2:**
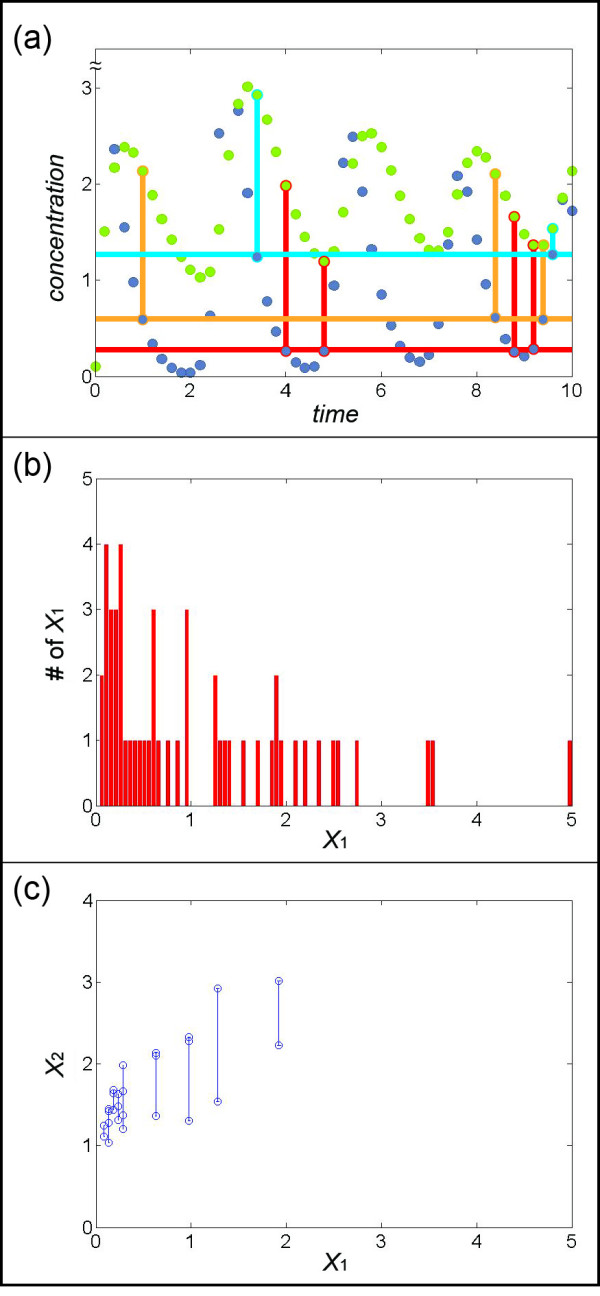
**(a) Fixing*X***_**1**_**within a narrow range (~0.26), four instances of*X***_**1**_**are found (solid red circles).** Fixing *X*_1_ within another narrow range (~0.6) provides three instances of *X*_1_ (solid orange circles). Similarly, two instances of *X*_1_ are found for *X*_1_ ~1.26 (solid blue circles). **(b)** Collection of 34 “bins” that exhibit the number of times *X*_1_ has approximately the same value given on the *x*-axis; the range of each bin was chosen as 0.05. Among the 34 bins, 9 bins have at least two instances of the same *X*_1_; all other bins are discarded. **(c)** Representation of different *X*_2_ values corresponding to at least two *X*_1_ values in each of the 9 remaining bins. The bars connect the two or more *X*_2_ values in each bin.

For instance, *X*_1_ has similar values (~0.26) at time points 4, 4.8, 8.8, and 9.2, while *X*_2_ has different values at these time points (Figure 2(a)).

We repeat this type of screening for different sets of the same or very similar values of *X*_*in*_. The result is a set of sets with equal *X*_*in_const*_ values within each set but different *X*_*in_const*_ values for different sets. These sets form a histogram with a bin for each *X*_*in_const*_. If the range of each bin is small enough, we can assume every *X*_*in*_ in the same bin to have very similar values, so that their corresponding *v*_*in_const*_ are also very similar. Henceforth, we only retain bins with at least two entries. An example in the illustrative example consists of time points 3.4 and 9.6, where *X*_1_ has again similar values. In this case, the value is ~1.26, which is different from the value we screened before. Similarly, for time points 1, 8.4, and 9.4, *X*_1_ has a value of ~0.6 (Figure 2(a)). Figure 2(b) shows many situations in the dataset where *X*_1_ has approximately some fixed value, and these sets of *X*_1_ are reflected in a “bin database of values.” Within each bin, the corresponding value of *v*_2*c*_ is very similar as well.

Suppose we have identified *P* bins that contain at least two *X*_*in*_. For these bins we determine the corresponding *X*_*out*_ values, which are typically different from each other. Suppose that bin *p* contains *q* values. Thus, we obtain *q* equations of the type

(10) $$S_{i}(bin_{p})=v_{in\_const}(bin_{p})-v_{\mathrm{out}}(bin_{p}),\;p=1,\ldots ,P\text{,}$$

where v_*in_const*_ (*bin*_*p*_) always has the same value, but *S*_*i*_ (*bin*_*p*_) and *v*_*out*_ (*bin*_*p*_) have different values. For our illustration we specify nine bins (*P* = 9) (which have at least two *X*_1_ (Figure 2(b)), and their corresponding values of *X*_2_ at the same time points are shown in Figure 2(c). The 6^th^ of the nine bins (shown as the orange bin in Figure 2(a)) contains three instances of *X*_1_. Therefore, we obtain three equations of the type

(11) $$S_{2}(bin_{6})=v_{2c}(bin_{6})-v_{3}(bin_{6})\text{.}$$

Equation (10) is formulated analogously for each bin *p* =1, …, *P*. In each case, *v*_*out*_ (*bin*_*p*_) can be represented as at least two equations of the type

(12) $$v_{\mathrm{out}}(bin_{p})=v_{in\_const}(bin_{p})-S_{i}(bin_{p})\text{,}\;p=1,\ldots ,P\text{.}$$

Since we do not know the functional form of *v*_*in*_, we do not know the numerical value of *v*_*in_const*_ (*bin*_*p*_). However, since *v*_*in_const*_ (*bin*_*p*_) is a constant for each bin, the relative positions of a group of values of *v*_*out*_ (*bin*_*p*_) depend on each value –*S*_*i*_ (*bin*_*p*_) within a given bin, and the slope values can be measured directly from the time series data. In addition, since *v*_*out*_ (*bin*_*p*_) is solely determined by *X*_*out*_ (*bin*_*p*_), we can characterize the relative positions of a set of *X*_*out*_ (*bin*_*p*_) and their corresponding values –*S*_*i*_ (*bin*_*p*_). Collecting these relationships, we can establish a plot of *X*_*out*_ (*bin*_*p*_) versus –*S*_*i*_ (*bin*_*p*_). If the bin contains only two points of *X*_*out*_, we consider them as a pair and link them with a connecting line. If the bin contains *q* points of *X*_*out*_ (where *q* > 2), we sort *X*_*out*_ based on their values and connect every two adjacent points as a pair to form a total of *q*-1 pairs. In order to address these pairs, we use an additional index for the position of each pair in each bin, such as (*X*_*out*_ (*pair*_*r*_)(1), –*S*_*i*_ (*pair*_*r*_)(1)) for the first point and (*X*_*out*_ (*pair*_*r*_) (2), –*S*_*i*_ (*pair*_*r*_) (2)) for the second point, where *r* = 1, …, *q*–1.

**Figure 3:**
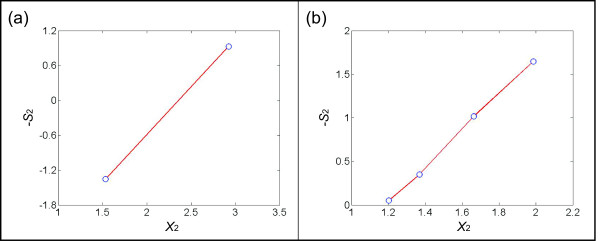
**(a) The 8**^**th**^**bin in Figure**2**(b) contains two different*X***_**2**_**values corresponding to the “blue” instances in Figure**2**(a) for*X***_**1**_**~1.26.** The corresponding values of *X*_2_ and –*S*_2_, obtained from the plot of *X*_2_ versus –*S*_2_, are (1.54, -1.35) and (2.93, 0.93). These two points are considered a pair and linked with a red line. **(b)** The 5^th^ bin of Figure 2(b), corresponding to the “red” instances in Figure 2(a), contains four instances of *X*_1_ ~0.26. Their corresponding values of *X*_2_ and –*S*_2_ are (1.20, 0.05), (1.37, 0.35), (1.66, 1.02), and (1.99, 1.65). Two points each are considered a pair and linked with a red line.

**Figure 4:**
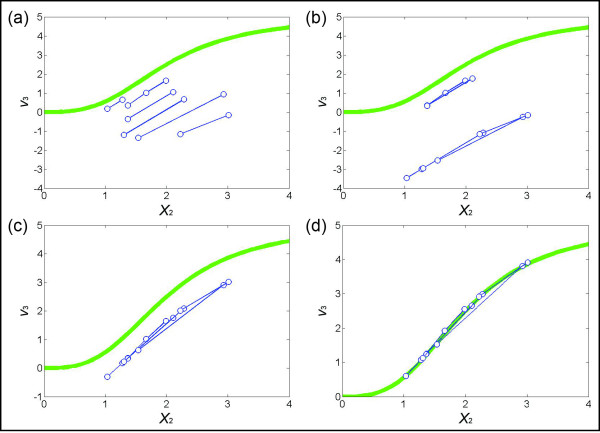
**(a) Pairs of points satisfying a threshold value of*d*(see*Methods*) greater then 0.2.** Seven pairs (*s* = 7; connected by blue lines) are selected for the following steps. The green line is the true functional representation of *X*_2_ versus *v*_3_, which in an actual situation is not known. **(b)** Pairs in (a) are merged, based on the distances between points in each “node” and the distances between two points in a pair. **(c)** Subgroups of pairs in (b) are merged. **(d)** If the value of *v*_3_ is known for *X*_2_ = 1 or for some other value. The entire cluster of lines is vertically shifted accordingly. If small values of *X*_2_ are covered by the pairs, the shift is determined by the observation that a flux is usually zero if the substrate concentration is zero. Here, the sum of errors between the estimated points and corresponding points on the true green line is 0.0354.

To continue the illustration, the 8^th^ bin contains two instances of *X*_1_ ~1.26. The corresponding values of *X*_2_ are 1.54 and 2.93, and the –*S*_2_ values are −1.35 and 0.93, respectively. The points in the plot of *X*_2_ (*bin*_8_) versus –*S*_2_ (*bin*_8_) are therefore represented as (1.54,–1.35) and (2.93, 0.93). We consider these two points as a pair and link them using a red line (Figure 3(a)). Similarly, the 5^th^ bin contains four instances of *X*_1_ ~0.26. Their corresponding values of *X*_2_ are 1.20, 1.37, 1.66, and 1.99, and the –*S*_2_ values are 0.05, 0.35, 1.02, and 1.65, respectively. The points in the plot of *X*_2_ (*bin*_5_) versus –*S*_2_ (*bin*_5_) are therefore represented as (1.20, 0.05), (1.37, 0.35), (1.66, 1.02), and (1.99, 1.65). Two points each are considered a pair and linked with a red line (Figure 3(b)). After the pairs of points are determined, we prune the set by neglecting pairs where the distance between *X*_*out*_ (*pair*_*r*_)(1) and *X*_*out*_ (*pair*_*r*_)(2) is below some threshold $d_{r}=\left|X_{\mathrm{out}}(pair_{r})(1)-X_{\mathrm{out}}(pair_{r})(2)\right|$. The reason is that small line segments tend to lead to unduly high estimation errors. The default value for *d*_r_ is set as 0.2 in the examples shown in this article, but it will generally depend on the accuracy and quantity of the data. The higher the value is, the fewer pairs will remain after filtration. However, as long as the remaining pairs cover most of the spectrum in the *X* axis, an increase in *d*_*r*_ might be preferable. Suppose *s* pairs remain after this filtering. Figure 4(a) shows the collective result for the illustration example.

Equation (12) indicates that *X*_*out*_ (*bin*_*p*_) and –*S*_*i*_ (*bin*_*p*_) differ by a constant, since we do not know the value of *v*_*in_const*_ (*bin*_*p*_). This fact translates into a constant vertical shift in the *y* direction for each pair of points. In other words, the relative *y* positions of the pair are preserved and the pair has to be shifted together by a yet unknown amount. While we do not know the size of the shift for each individual pair of points, all points collectively represent the graph of *X*_*out*_ versus *v*_*out*_, and it is reasonable to assume that this graph is continuous and usually even monotonic. Therefore, the next step is to merge the individual pairs by determining a proper shift for each pair.

Intuitively, it is easy to see how to shift all pairs so that they are close to one continuous line. Automation of the process requires an algorithm that is not quite straightforward, but can be facilitated with a graphical user interface; technical details of a possible merging process are presented in Figure S1 of the Additional file 1. A pseudo-code of the merging is the following:

**SET** each pair of points as a node

**SET** each node as a subgraph

**WHILE** the graph is not connected

 **FOR** each subgraph in the graph

  **FOR** each node in the current subgraph

   **SET** other-subgraphs as the subgraphs; exclude the current subgraph

   **CALCULATE** the distance from the current node to every node contained in other-subgraphs

  **END FOR**

  **FIND** the shortest distance and its corresponding nodes

  **CONNECT** these two nodes

 **END FOR**

END WHILE

 

When the merging is completed, all pairs of points are close to a relatively smooth line, but the overall shift of the group of pairs is not known. We do know that essentially all metabolic fluxes will have values close to zero when their substrate concentration approaches zero. Thus, if sufficiently small substrate values are available in one of the bins, one easily estimates a reasonable shift. Should the flux value associated with some substrate concentration be known, the shift can be determined from this information. A further alternative is the following. If the inferred trend line suggests that the flux follows some rate law, such as a Hill function, the parameters of this function, together with the appropriate shift, can be obtained in a single optimization step.

Figure 4(b) shows, for the illustrative example, the process of merging and shifting. The human eye has no problem accomplishing this task intuitively. In the automated process (see Additional file 1), one connects each “node” (pair of points) with its closest-neighbor node and positioning the pair of points. This process creates two sub-groups of points. We recalculate the distance between each node in a sub-group with the nodes in the other sub-group, determine the closest pair of nodes, connect them, and shift the corresponding pairs into one sub-group as shown in Figure 4(c). Suppose the value of *v*_3_ is known for *X*_2_ = 1. If so, we ultimately shift the entire trend accordingly. The result is shown in Figure 4(d). A shift based on the association between zero flux and zero substrate concentration is an alternative, although it does not uniquely prescribe a solution in this case.

Finally, based on the numerical or graphical flux profile thus determined, one may test candidate functions that capture the flux-substrate relationship. For instance, the result in the illustrative example shows that the functional relationship of *X*_2_*vs. v*_3_ is *s*-shaped. It could thus be consistent with the (true) Hill function in Eq. (4), although the computed result itself certainly would not prove that this format is correct. If one assumes, based on the results, that a Hill function is appropriate, one may fit this functional form to data to find the optimal parameter values of the flux-metabolite dependency. Without making such an assumption, one may alternatively connect the dots in Figure 4(d) with a continuous line and interpolate the values of fluxes using a spline or another smoothing method. The resulting trend line can be used as a “look-up” plot.

Now that we have determined *v*_3_, it is easy to compute *v*_2_ from the measured slopes of *X*_2_. The plot of *v*_3_ is slightly curved, which is consistent with its power-law function in Eq. (4), although again, there is no proof. The *Results* section discusses further examples.

**Figure 5:**
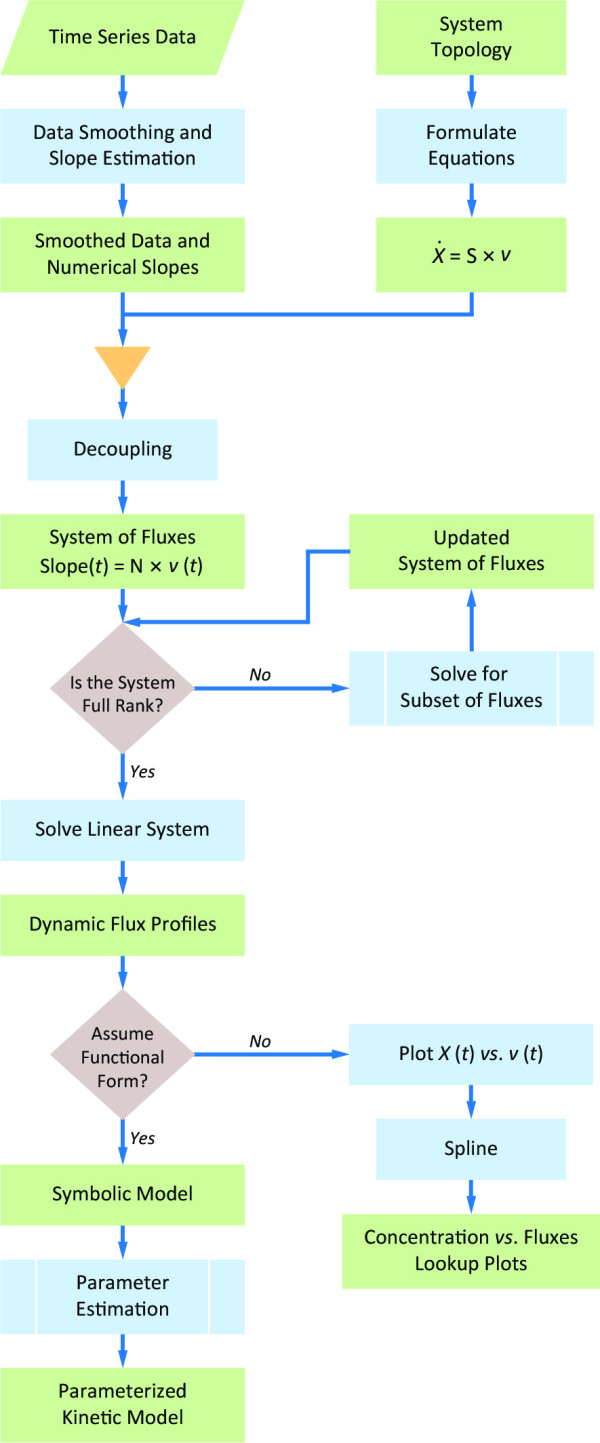
**Flowchart of the proposed method.** Starting with experimental time series, the data are smoothed and balanced for mass conservation, if necessary. The slopes of the time series at each time point are estimated. Combined with the knowledge of the system topology, substitution of the derivatives in the ODE with slope information yields a linear system of fluxes. If the system has full rank, solve the system with techniques from linear algebra. If the system is underdetermined, use auxiliary steps, as proposed in this article, to solve a subset of the fluxes until the system is of full rank. The results are the dynamic profiles of all extra- and intra-cellular fluxes in the system. If desired, make assumptions regarding the functional forms of the fluxes. These functions correspond to symbolic flux representations that can be independently fitted to the respective dynamic flux profiles and result in a fully parameterized kinetic model. As an alternative each process may be approximated as a piecewise function, for instance using spline methods.

The parameters of any candidate functional form are easily estimated, because no differential equations are involved and the problem is of low dimension; they represent a fully parameterized kinetic model for the flux term itself and, subsequently for the differential equation. Due to this simplicity, it is even possible to scan a variety of candidate functions and assess their appropriateness. If a suitable functional format can be determined with appropriate parameter values, the task is completed. If not, one may represent the flux-substrate plot with a piecewise-polynomial function, such as cubic spline. Even in this non-explicit, numerical format, the result is sufficient to reduce one or two degrees of freedom in the overall DFE task. Figure 5 presents the overall flow and concept of the method.

The procedure described above has generated one or two additional flux estimates. For the example in Eq. (3), the determination of *v*_2_ and *v*_3_ “fills” the rank, and the system can be uniquely solved. In fact, only one of the two is needed. For examples where one or two additional fluxes are not sufficient for a unique solution, the same procedure has to be performed with other equations until enough fluxes are determined to make the flux system full rank. DFE subsequently identifies all other fluxes as plots against time or against their substrates and modulators.

In cases where fluxes contain more than one variable, the time courses have to be screened for combinations where the contributing variables have the same values. The concepts of the procedure are exactly the same as for the univariate case, but the implementation is obviously more involved (see Additional file 1). Also, such combinations are rarer than in the cases described above, so that these situations require more diverse datasets for structure identification.

## Results

The simple linear pathway shown in the previous section illustrated the concepts of the proposed extension to DFE. This section describes applications of the proposed methods in the context of further didactic and actual examples that become increasingly more complicated. We begin with two artificial cases with distinct characteristics and conclude with the analysis of experimental observations describing trehalose metabolism in the yeast *Saccharomyces cerevisiae*.

### Branched pathway with feedforward activation and feedback inhibition

**Figure 6:**
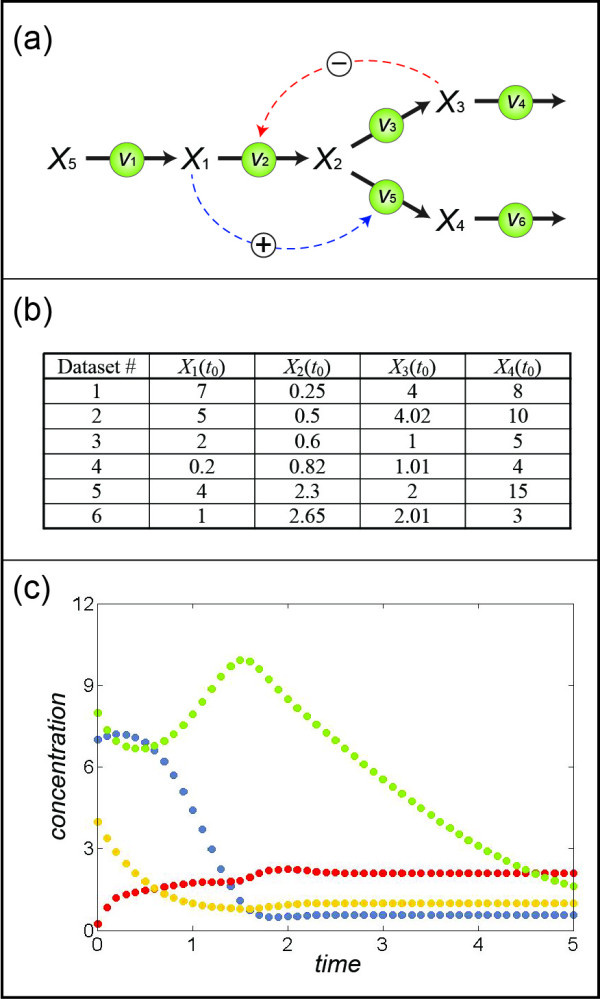
**(a) Metabolic network with positive feedforward and negative feedback.** All enzymatic reactions are assumed to follow Michaelis-Menten or Hill kinetics except for those corresponding to *v*_2_ and *v*_5_, which are assumed to be represented with an Irreversible General Hyperbolic Modifier Kinetic function and with an Irreversible Hill function with one modifier, respectively (see Eq. (14) for details). **(b)** Sets of initial conditions used to generate six different datasets. **(c)** Time series data corresponding to the first set of initial values in (b); *X*_1_, *X*_2_, *X*_3_, *X*_4_ are represented by blue, red, orange, and green dots, respectively.

Consider a branched pathway with fluxes represented by various functional forms, including Michaelis-Menten and Hill functions with inhibition and activation. The pathway, shown in Figure 6(a), can be described by the following set of ordinary differential equations [30]:

(13) $$\begin{array}{l} {\dot{X}}_{1}\text{ = }v_{1}-v_{2}\\ {\dot{X}}_{2}\text{ = }v_{2}-v_{3}-v_{5}\\ {\dot{X}}_{3}\text{ = }v_{3}-v_{4}\\ {\dot{X}}_{4}\text{ = }v_{5}-v_{6} \end{array}$$

The kinetic descriptions for each of the reactions are:

(14) $$\begin{array}{l} v_{1}=\frac{16\times X_{5}}{1+X_{5}}\\ v_{2}=\frac{65\times \frac{X_{1}}{0.3}\times \left(1+\frac{0.04}{2.5}\times \frac{X_{3}}{0.12}\right)}{1+\frac{X_{3}}{0.12}+\frac{X_{1}}{0.3}\times \left(1+\frac{X_{3}}{2.5\times 0.12}\right)}\\ v_{3}=\frac{5\times {\left(X_{2}\right)}^{4}}{5+{\left(X_{2}\right)}^{4}}\\ v_{4}=\frac{8\times X_{3}}{1+X_{3}}\\ v_{5}=\frac{58\times {\left(\frac{X_{2}}{11}\right)}^{3}}{{\left(\frac{X_{2}}{11}\right)}^{3}+\frac{1+{\left(\frac{X_{1}}{1}\right)}^{3}}{1+63\times {\left(\frac{X_{1}}{1}\right)}^{3}}}\\ v_{6}=\frac{8\times X_{4}}{1+X_{4}} \end{array}$$

As before, we use these formats to generate artificial data, but subsequently assume no knowledge of their characteristics.

The system in Eq. (13) is not of full rank. Thus, some of the fluxes need to be determined with the proposed method. For our illustration, we select the third equation in Eq. (13), because it contains only two fluxes; also, *v*_3_ depends only on *X*_2_, and *v*_4_ depends only on *X*_3_, which we know from the topology of the pathway. In the previous example, all time series were oscillating and it was easy to find enough data points where one variable is fixed and other variables display different values. In the present example, each single dataset displays changes over time that show few repeated concentration values (see Figure 6(c)). In such a situation, which is more typical than the earlier illustration example, one can use exactly the same method, applied to multiple datasets with different dynamic profiles, as long as one can validly assume that the functional forms are not affected by differences among the datasets.

**Figure 7:**
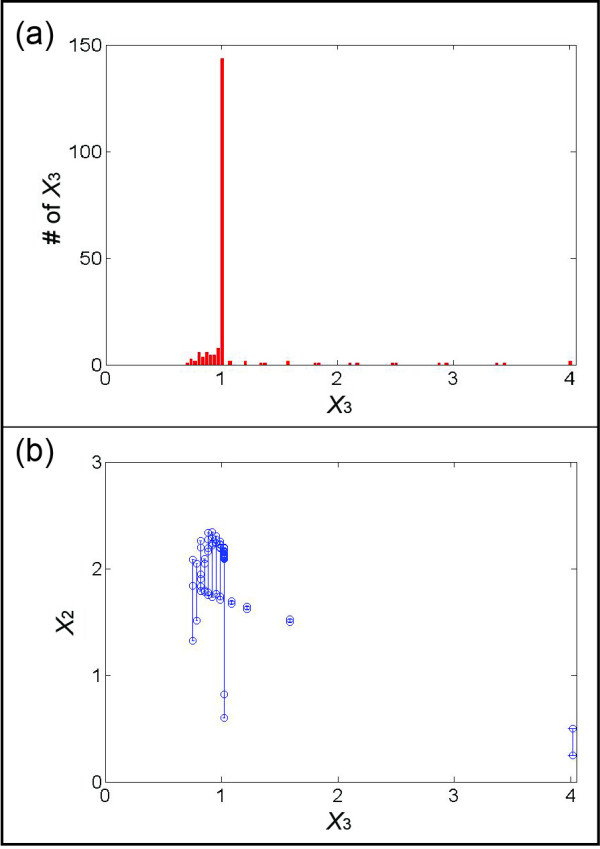
**(a) Bins of instances of*X***_**3**_**for different values; the range of each bin is chosen as 0.033.** Among the 26 bins, 13 bins have at least two *X*_3_ values; the others are discarded. **(b)** Representation of 13 sets of corresponding *X*_2_ values in those bins that have at least two *X*_3_. The bars connect two or more *X*_2_ values within each *X*_3_ bin.

**Figure 8:**
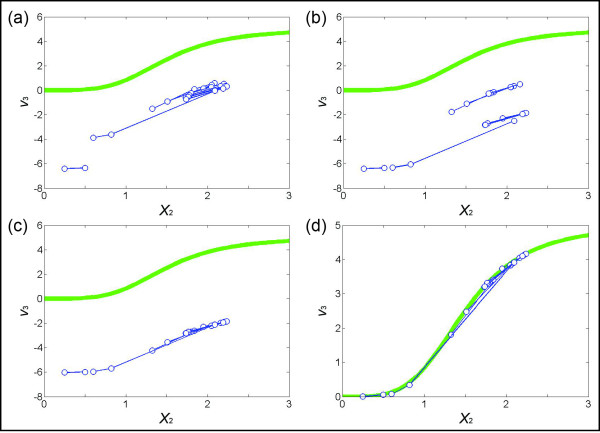
**(a) Collection of*s*pieces exceeding a chosen threshold*d*(here*s*** **= 12 and*d*** **= 0.2; see*Text*).** The green line is the “true” functional representation of *X*_2_ versus *v*_3_. **(b)** Pairs in (a) are merged based on their distances and on the distances between two points in a pair. **(c)** The subgroups of pairs in (b) are merged. **(d)** The sigmoidal shape of points in (c) suggests that the flux of the smallest *X*_2_ (~0.25) should be close to zero. The sum of errors between the estimated points and their corresponding true values (on the green line) is 0.0551.

For this illustration, we simulated multiple datasets with the initial values presented in Figure 6(b), which could easily reflect actual experimental settings. Figure 7(a) shows the result of binning *X*_3_ by using the first four datasets in Figure 6(b). The corresponding pairs of *X*_2_ (Figure 7(b)) range from 0.25 to 2.24, which covers most of the range of *X*_2_ in the four datasets (from 0.25 to 2.34). The merging process of pairs is shown in Figure 8, with panels corresponding to those in Figure 4. In particular, Figure 8(c) exhibits the merged points, which evidently form a sigmoidal shape where the first few points are basically flat. Therefore, one can assume the flux at the smallest *X*_2_ (~0.25) to be close to zero and shift the entire set of merged pairs up by about six units to obtain the estimates of *v*_3_. Indeed, this step recoups the true flux, which is shown in green, but would be unknown in a real application. Once *v*_3_ is determined, the system of Eq. (13) is still underdetermined and another flux needs to be estimated to make the system full rank. The most straightforward choice is *v*_4_, which is directly computed from *v*_3_ and the measured slopes of *X*_3_.

Instead of *v*_4_, one could also estimate an additional flux from another equation in Eq. (13) using the same procedure, for example, by solving *v*_5_ and *v*_6_ in the fourth equation. Flux *v*_6_ depends only on *X*_4_ but *v*_5_ depends on two variables *X*_1_ and *X*_2_. The steps of estimating *v*_5_ and *v*_6_ are described in Additional file 1. We also tested the proposed method by using six datasets in Figure 6(b) and the results similarly recover the true functional form (data not shown).

The proposed method was also tested on a five-variable system that has been used as a benchmark problem in many articles (*e.g.*[24,31-33]). To demonstrate the applicability of the method, we also added artificial noise to the time series data in this example and randomly picked sub-datasets from data generated with ten conditions. The details and results are shown in Additional file 1.

### Glycolysis and trehalose production

**Figure 9:**
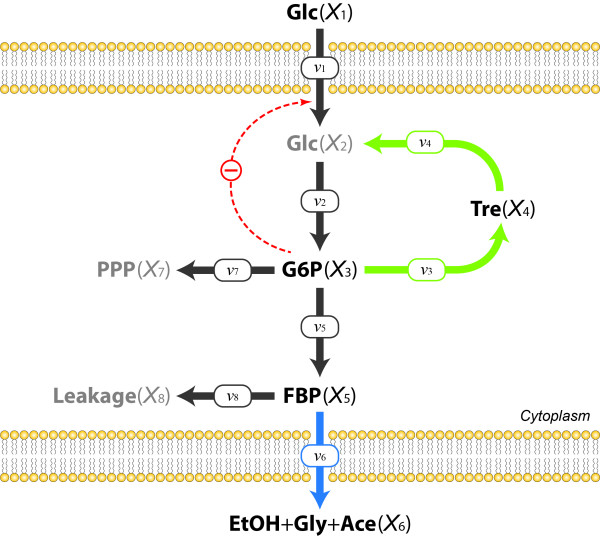
**Schematic representation of a simplified model of glycolysis and the trehalose cycle in the yeast*Saccharomyces cerevisiae*(adapted from**[34]**).***X*_*i*_ and *v*_*i*_ represent dependent variables and fluxes, respectively. One inhibitory interaction is shown in red. Abbreviations: *X*_1_, extracellular glucose; *X*_2_, intracellular glucose; *X*_3_, glucose 6-phosphate; *X*_4_, trehalose; *X*_5_, fructose 1,6-bisphosphate; *X*_6_, extracellularly accumulating end products (ethanol, glycerol and acetate); *X*_7_, mass diverted into the pentose phosphate pathway; *X*_8_, mass consumed by other pathways (*e.g.*, TCA); *v*_1_, glucose transport; *v*_2_, hexokinase and glucokinase; *v*_3_: aggregated step of all enzymatic steps between glucose 6-phosphate and the production of trehalose; *v*_4_, trehalase; *v*_5_, phosphoglucose isomerase and phosphofructokinase; *v*_6_, aggregated step of all enzymatic steps between fructose 1,6-bisphosphate aldolase and the release of end-products; *v*_7_, flux into the pentose phosphate pathway; *v*_8_, flux towards other pathways (leakage). Metabolites without available experimental measurements are shown in gray. The flux *v*_6_ (blue) is directly measurable from the time series of *X*_6_. Fluxes *v*_3_ and *v*_4_ (green) were estimated using the proposed method.

**Figure 10:**
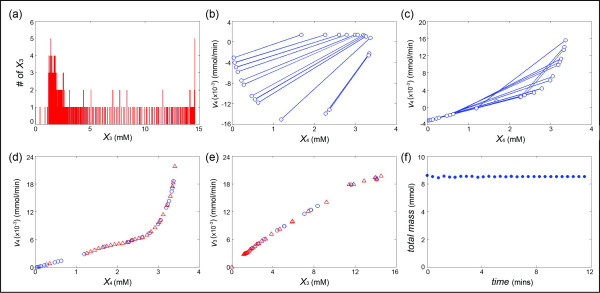
**Experimental metabolite time courses of glucose metabolism determined by*in vivo***^**13**^ **C-NMR in*Saccharomyces cerevisiae*grown under optimal temperature (30°C) with a single pulse of glucose (65 mM) (adapted from**[34]**).** The dots for *X*_1_, …, *X*_6_ are experimental measurements, while *X*_7_ was determined from the flux *v*_7_, which was inferred with the methods described in the *Text*. The lines are the result of a model simulation with the inferred fluxes. The end products (ethanol, glycerol and acetate) are summarily represented as *X*_6_ .

This last example describes in a simplified fashion how the baker’s yeast *Saccharomyces cerevisiae* converts glucose into end products and how trehalose is synthesized and degraded in a cyclic pathway (Figure 9). The data [34] consist of actual *in vivo* NMR measurements of metabolic profiles that characterize how the yeast responds to heat stress in two time regimes at the genome, protein, and metabolic levels. For the illustration here we use the metabolite dynamics of normally grown cells that were then exposed to heat stress (39°C) and fed with a pulse of glucose. Immediately after glucose addition, the initial metabolite pools (G6P and FBP) increase, while trehalose (Tre) increases with a short delay and begins to decrease slightly after two minutes. The end products ethanol, glycerol, and acetate gradually accumulate. The concentration data are shown as dots in Figure 10, together with the modeling results that are described next.

The model contains eight dependent variables and eight fluxes, as shown in Eq. (15), where *V*_ext_ and *V*_int_ represent the extracellular (0.05 L) and intracellular (0.00717 L) volume of the bioreactor and the cell population, respectively. Each of the fluxes is a function of some of the variables, as shown in Eq. (16), but it is important to note that we do not make any assumptions regarding the functional forms of the fluxes. In principle, DFE seems to be directly applicable. However, the time series data contain the measurements of only five of the metabolites, namely Glc (*X*_1_), G6P (*X*_3_), Tre (*X*_4_), FBP (*X*_5_), and extracellularly accumulated end products (EtOH, Gly, and Ace; *X*_6_). Without the measurements of *X*_2_, *X*_7_, and *X*_8_, the system in Eq. (15) is not of full rank and, due to the experimental set-up, *v*_7_ and *v*_8_ cannot be measured or determined directly by estimating slopes.

To complement the rank of the flux system, we use the proposed method of flux estimation. First, one should note that the measurements of Glc (*X*_1_) concern extracellular glucose. Thus, *X*_1_ is easy to measure experimentally, but it is very difficult to obtain good measurements of intracellular glucose (*X*_2_), because it is immediately converted in to G6P (*X*_3_). Thus, the proportion of Glc (*X*_2_) is negligible in comparison to Glc (*X*_1_), and because the measured concentration of glucose is close to the sum of Glc (*X*_1_) and Glc (*X*_2_), we merge *X*_1_ and *X*_2_ into one pool, which is represented by the sum of the first two equations in Eq. (15). Furthermore, the amount of material entering the pentose phosphate pathway (PPP; *X*_7_) is not directly measurable, but independent lab experiments had indicated that it has a value of approximately 5% of the glycolytic flux; thus $v_{7}=0.05\times v_{5}$[34]. With these simplifications, the system can be formulated as:

(15) $$\begin{array}{l} {\dot{X}}_{1}\text{=}-v_{1}/V_{\mathrm{ext}}\\ {\dot{X}}_{2}\text{=}(v_{1}+2v_{4}-v_{2})/V_{\mathrm{int}}\\ {\dot{X}}_{3}\text{=}(v_{2}-2v_{3}-v_{5}-v_{7})/V_{\mathrm{int}}\\ {\dot{X}}_{4}\text{=}(v_{3}-v_{4})/V_{\mathrm{int}}\\ {\dot{X}}_{5}\text{=}(v_{5}-v_{6}-v_{8})/V_{\mathrm{int}}\\ {\dot{X}}_{6}\text{=}2v_{6}/V_{\mathrm{ext}}\\ {\dot{X}}_{7}\text{=}v_{7}/V_{\mathrm{int}}\\ {\dot{X}}_{8}\text{=}v_{8}/V_{\mathrm{int}} \end{array}$$

(16) $$\begin{array}{l} v_{1}=F_{1}\left(X_{1},X_{3}\right)\\ v_{2}=F_{2}\left(X_{2}\right)\\ v_{3}=F_{3}\left(X_{3}\right)\\ v_{4}=F_{4}\left(X_{4}\right)\\ v_{5}=F_{5}\left(X_{3}\right)\\ v_{6}=F_{6}\left(X_{5}\right)\\ v_{7}=0.05\times v_{5}\\ v_{8}=F_{8}\left(X_{5}\right) \end{array}$$

**Figure 11:**
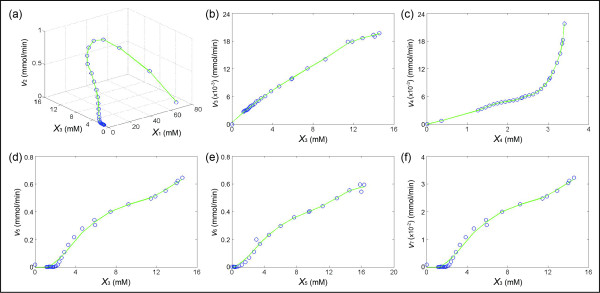
**(a) The experimental concentration data (29 time points) were smoothed and interpolated with a spline function, thereby yielding metabolite levels of*X***_**3**_**at about 300 time points.** These *X*_3_ values were put into 186 different bins with size 0.03. Among these, 54 bins have at least two *X*_3_ values. **(b)** Graph of *X*_4_ values, corresponding to at least two *X*_3_ values in each of the *X*_3_ bins. Selection of *s* pairs with a threshold *d* of 0.3 (here *s* = 12; see *Text*) are selected. **(c)** Pairs in (b) are merged. **(d)** Resulting functional plot of *X*_4_*vs. v*_4_; the blue dots represent the dots in (c), while the red triangles represent the true plot of *X*_4_*vs. v*_4_ in the dynamic model; in reality, these would not be known. **(e)** Functional plot of *X*_3_*vs. v*_3_ (blue dots), calculated from the blue dots in (d), and true values of *X*_3_ (red triangles) according to the dynamic model. **(f)** Confirmation that the total mass (represented as the number of 3-carbon units) does not change appreciably over time.

To supplement the underdetermined DFE, we select the equation ${\dot{X}}_{4}=(v_{3}-v_{4})/V_{\mathrm{int}}$ in Eq. (15), since it contains only two terms and the measurements of *X*_3_ and *X*_4_ are available. As before, we fix *X*_3_ at some values (Figure 11(a)) and find the corresponding *X*_4_ and –*S*_4_ (Figure 11(b)). The merged pairs suggest an approximately exponential function (at least for the range of available *X*_4_) and the minimum of *X*_4_ is very close to zero. For a concentration close to zero, the value of the flux should be close to zero as well. Therefore, the entire cluster of pairs is moved up around 4 units, and the updated functional plot is shown in Figure 11(d). The corresponding *v*_3_ can now be calculated accordingly and transformed to the form as fluxes versus time. After the determination of *v*_3_ and *v*_4_, the system of Eq. (15) becomes full rank and the rest of the fluxes at each time point can be solved with DFE even without knowledge of the times series of *X*_7_ and *X*_8_. Indeed, the time courses of *X*_7_ and *X*_8_ can be calculated via point-by-point integration of *v*_7_ and *v*_8_. Upon the determination of the concentrations of all variables, the total mass over time can be calculated, confirming no significant loss or gain of mass (Figure 11(f)).

**Figure 12:**
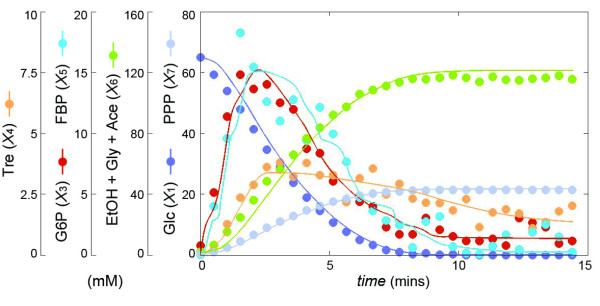
**Results from the proposed method and subsequent application of DFE to yeast data from the model in Figure**9**.** Shown are metabolite concentrations against fluxes at different time points (blue dots), connected by inferred trend lines for all fluxes (green lines).

Once we have obtained the time series of all fluxes, we can generate the plots of concentrations of metabolites that are involved in the enzymatic reactions (see Eq. (16)) versus a flux. The results are shown in Figure 12. As a validation test, we used these numerical flux representations of each enzyme catalyzed reaction to simulate the concentration changes of metabolites. The simulation results are shown in Figure 10.

## Discussion

Of all steps in the generic mathematical modeling process, parameter estimation and structure identification continue to be among the most severe bottlenecks for modeling biological systems. Until recently, this task was typically pursued from the bottom up by using local data from individual enzymatic steps. However, modern techniques of molecular biology have provided us with a strikingly different estimation strategy, namely a top-down or inverse approach, which is based on dynamic time series data that are being generated with rapidly increasing frequency and quality. Many recent articles have proposed various methods to tackle this inverse estimation problem using time series data. However, none of these methods are effective in all cases. Furthermore, almost all methods have been focusing on the goodness of fit and the speed of the algorithm, but not necessarily the quality of fit in terms of the validity of the model, extrapolation ability, and predictive power with respect to data not used in the estimation. In addition, there has been little discussion of the diagnostic tools for data fits beyond the residual error. For instance, it is possible that a fit is good in terms of the residual error, but that the estimated fluxes are incorrect because of numerical compensations between terms within the model [10].

Dynamic Flux Estimation (DFE) [10] addresses several of these issues successfully, but only if the data are rather comprehensive. More limiting, DFE requires that the system of fluxes is of full rank. When the number of fluxes exceeds that of the dependent metabolites, either because of the stoichiometry of the pathway or due to the lack of measurements of some metabolites, DFE cannot be applied directly, because the system of fluxes is underdetermined. To supplement such a system, we recently proposed methods for supplementing DFE with other information that may be used as a substitute for unknown fluxes [19]. However, these methods are successful only under certain restrictive conditions, for instance, when the enzymes in the system are well characterized under pertinent conditions, sufficient kinetic information is available, and all significant metabolic time series are measured. One could also determine some of the fluxes within the system by fitting pre-selected models to time series data. However, this pre-selection requires the definition of functional forms for the reactions in question, which in truth are often unknown.

In this article we propose a model-free approach with minimal assumptions to supplement DFE with information already embedded in the time series data. The proposed method starts with the selection of a decoupled equation; preferable one that contains a minimal number of terms and contributing metabolites. Within this equation, we repeatedly fix one or a few variables that have constant or very similar values within certain small ranges, and find the corresponding values of the variables that appear in another flux of the equation. The result of this step is a plot of a flux versus a metabolite, with several pairs of points showing the relative positions of the true metabolite concentration and the flux values in each pair. The position of each pair is initially subject to shifting in the *y* direction by an unknown amount. The correct shifting of pairs may be accomplished with an automated or manual merging process that, for instance, accounts for the fact that the flux value should be zero when the metabolite concentration is zero. One could also measure the flux value at some metabolite concentration experimentally. Furthermore, if an enzymatic rate function is deemed correct and corresponding kinetic information is available, the vertical shift in the flux can be calculated. Once the metabolite-flux plots are established for all fluxes, one can select a suitable mathematical representation for the entire dependency or use a piecewise approximation for different ranges of data. One could also use the metabolite-flux relationships directly as “look-up” plots.

The proposed method may appear cumbersome or even baroque. However, one should consider that it solves a problem that so far has not even been addressed—let alone solved—with any systematic approach. Also, the method is presently likely to suffer from a lack of suitable data. But judging by the development of high-throughput experimental methods and the number and increasing quality of published time series over the past decade, this issue seems to be primarily a matter of time. Indeed, one should expect that it will soon be feasible to generate strategically selected, multiple datasets for the identification of a system, which differ slightly in their settings. These datasets must come from experiments that do not alter the functional characteristics of the fluxes in the system but might, for instance, measure system responses under modestly different substrate or inhibitor conditions. At the same time, the data should be representative of the dynamics of the system within the pertinent ranges of its variables.

The method involves one step that is subject to bias. Namely, the overall shifting of the flux-metabolite relationship requires extrapolation or some other information, unless metabolite concentrations close to zero are available. To resolve this issue, it might be possible to determine a reference point for the shift from enzymatic or kinetic information. However, in many cases, this information will have been obtained *in vitro* and possibly under different conditions. A more direct approach would be to measure a flux value experimentally at some point, for instance, at the steady state. Such a measurement is relatively simple when the flux of interest is an input or output flux. It might also be possible to measure some fluxes directly by estimating the rate of consumption and production of the initial substrate or the end product, respectively. However, the measurements of fluxes at these locations are usually of lower interest since they are seldom associated with the underdetermined subsystems of the internal fluxes. One or more intracellular fluxes could also possibly be characterized through measurements of a suitable isotopomer distribution at steady state (*i.e.*[17,35]), but such data are still rare. Finally, if one has valid reason to assume a particular format for a flux, such as a Michaelis-Menten or Hill function, the shift may be obtained through optimization. All estimation methods are negatively affected by noise, and the proposed method is no different (see Additional file 1). However, issues of noise can be logistically separated from this method to some degree. Namely, the time series data used as basis for the proposed method may (in fact, should) be smoothed in a preprocessing step, for instance with a filter [2,26,27]. This well-established step allows an assessment of the characteristics of the noise as well as the smoothing process itself. Once the data are smoothed and noise is thus reduced, the proposed method is applied as if the data had been noise free.

Outside these remaining details, the proposed method has several notable advantages. First, no assumptions are needed regarding the mathematical representations when determining the individual fluxes. Second, the application of the method is not limited to a small range of a metabolite or its flux. Instead, it allows the modeler to examine the full spectrum of the functional form, depending on how widely the available time series data cover metabolite concentrations along the *x* axis of the metabolite-flux plot. Third, even under the condition that some of the time series are missing, the proposed method can still recover—at least to some degree—the governing flux profiles. Finally, since the range of coverage depends on the available datasets, we can, arguably for the first time, estimate how many data points are necessary to identify the functional format of a flux or what values of metabolite concentrations are needed to cover the concentration range of interest. Namely, if it is possible to implement the proposed method for a system at hand with sufficient reliability, then we know that we have enough data to assess the range over which the flux can be determined. If a wider range needs to be known, additional data will have to be made available in that extended range. This insight in itself will aid the design of specific experiments that can be used to generate more extensive functional plots.

## Conclusions

In this article we propose a systematic strategy to supplement and ameliorate the limitations of the method of Dynamic Flux Estimation (DFE). The proposed strategy makes no *a priori* assumptions regarding the model representation and uses instead information embedded in the time series data. The results demonstrate that the proposed approach successfully complements DFE in various situations. The method permits the examination of a full spectrum of functional forms, as well as a determination of whether at all, to what degree, or within what range, the available time series data can be validly represented in a particular functional flux format within a pathway model. Based on these results, one can, arguably for the first time, estimate how many data points are required to identify the functional format of processes within a system model and design experiments to generate data points that genuinely add new information to the parameter estimation and structure identification tasks.

## Competing interests

The authors declare no competing interests.

## Authors’ contributions

Ideas and concepts were jointly discussed among both authors. ICC developed and implemented the project under the supervision of EOV. Both authors contributed to the writing of the manuscript. Both authors read and approved the final manuscript.

## Supplementary Material

Additional file 1 **This file contains: (1) details regarding the process of merging pairs of points; (2) the estimation procedure for a four-variable branched pathway and results of two cases where fluxes contain more than one variable; and (3) the results of the method for a five-variable system where different levels of artificial noise were added to the time series data and sub-datasets were randomly picked from data generated with ten sets of initial conditions.**[24,31-33]. Click here for file
