# Supplementary material for: Estimation of dynamic flux profiles from metabolic time series data
Source: BMC Syst Biol. 2012 Jul 9;6:84. doi: 10.1186/1752-0509-6-84 (PMC3495652; doi:10.1186/1752-0509-6-84)
Supplement: Additional file 1 — This file contains: (1) details regarding the process of merging pairs of points; (2) the estimation procedure for a four-variable branched pathway and results of two cases where fluxes contain more than one variable; and (3) the results of the method for a five-variable system where different levels of artificial noise were added to the time series data and sub-datasets were randomly picked from data generated with ten sets of initial conditions.[24,31-33]. [file 1752-0509-6-84-S1.docx]

**System Estimation from Metabolic Time Series Data**

**(Additional File 1)**

I-Chun Chou and Eberhard O. Voit

**Process of merging pairs of points**

We consider each pair as a node in a graph and set as our goal to make the graph connected. The criteria of connecting two nodes are based on the distance between the points in each node and the length of the connecting line. In the first run of merging, each node finds its closest neighbor node and connects with it. The result of this procedure may already be a connected graph or consist of several subgraphs. In the latter case, repeat the procedure by calculating the distance between each node in a subgraph to the node in other subgraphs and connect the nodes with the shortest distance. Repeat the steps until the graph is connected. When the merging is accomplished, each pair (node) is shifted accordingly (see Fig. S1 and its legend for details). As an alternative, it might be possible to design a smoothing algorithm that directly merges the pairs so that the resulting trend exhibits minimal fluctuations.

**Branched pathway with feedforward activation and feedback inhibition**

This example shows two approaches to utilizing the fourth equation () of the branched pathway in Eq. (13) by fixing different variables. In this equation, *v*_5_ depends on two variables, *X*_1_ and *X*_2_, and *v*_6_ depends on *X*_4_. We look for different scenarios in datasets 1~4 in Fig. 6(b), where *X*_1_ and *X*_2_ have some fixed values *X*_1c_ and *X*_2c_. Again assuming that the flux is a function in the strict mathematical sense, we can deduce that *v*_5_ is also a constant with some value *v*_5c_. Thus, for the set of all *X*_1_ $\approx$ *X*_1c_ and *X*_2_ $\approx$ *X*_2c_ the equation of has the form

. (S1)

Next we find *X*_1_ and *X*_2_ which have fixed values in the dataset and put these *X*_1_ and *X*_2_ into each “grid” base on their values (Fig. S2). Note that this two-dimensional grid corresponds to the one-dimensional bins for fluxes in the text. Within each grid we find the corresponding *X*_4_ and merge the points using the same approach as described in *Text*. The results are shown in Fig. S3.


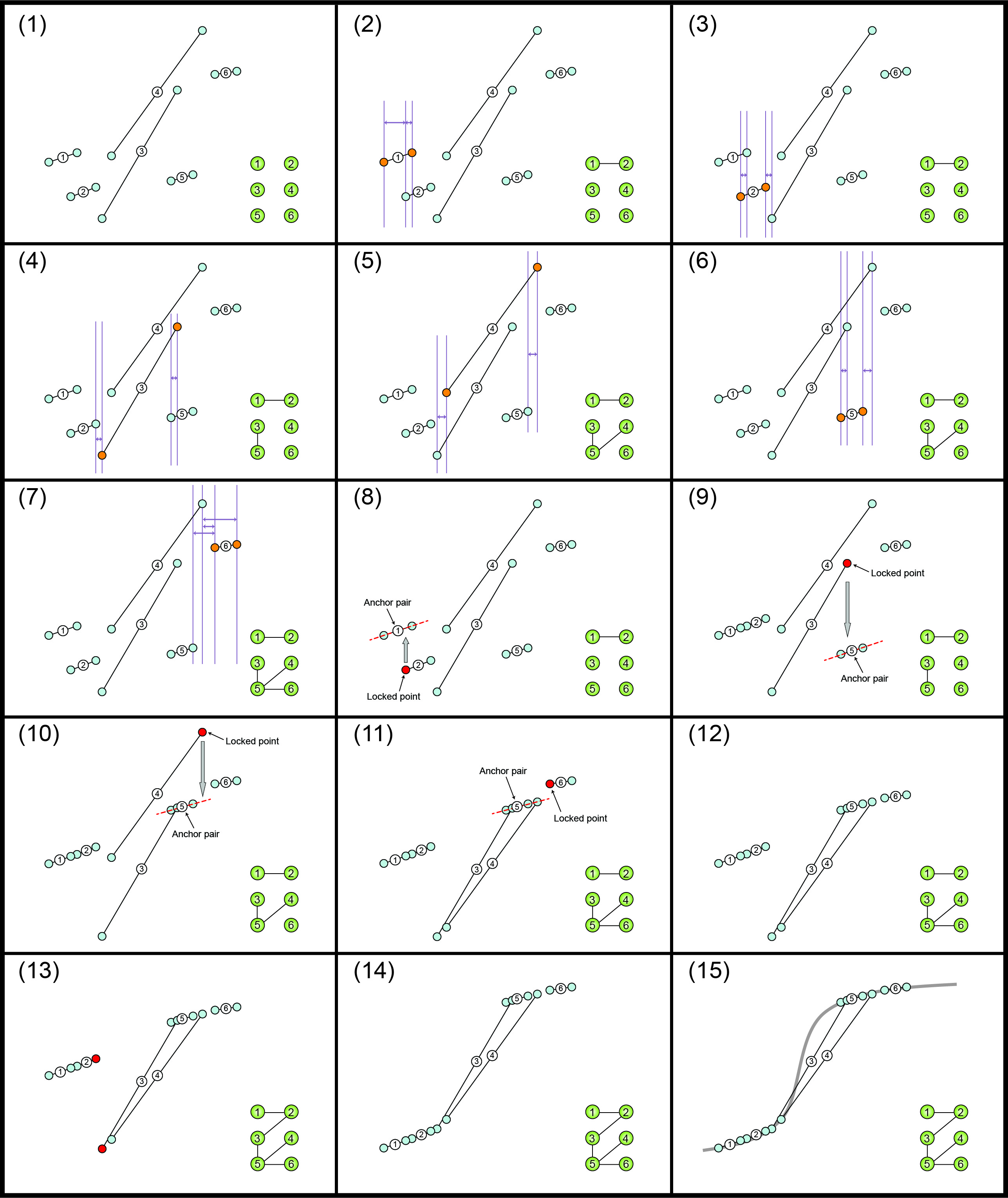


Figure S1. Details of the process of merging pairs of points. (1) Six pairs are identified, each of which is represented as a node. (2) Compute the distance of node ➀ and the rest of the nodes in *x*-direction. The right point of node ➀ has the shortest distance to the left point of node ➁. Node ➀ and node ➁ are therefore connected. (3) Compute the distance of node ➁ and the rest of the nodes. The distance from the left point of node ➁ to the right point of node ➀ is similar to the distance from the right point of node ➁ to the left point of node ➂. However, the length between the two points in node ➀ is shorter compared to node ➂; therefore, node ➁ is marked as connected to node ➀. (4) Similar to step (3), node ➂ is connected to node ➄. (5) Node ➃ is connected to node ➄. (6) Node ➄ is connected to node ➂. (7) Even though the distance between node ➅ and node ➄ is shorter than to node ➃, node ➅ is connected to node ➄ since the length between two points in node ➄ is much shorter. The six nodes form two sub-graphs. (8)-(11) Shift the pairs based on the steps from (2) to (7). For each pair of connections, the shorter piece (shorter distance between two points in the pair) is the “anchor pair” to which the corresponding pair will merge. Whichever point of the corresponding pair has a smaller distance between either point in the anchor pair, is designated as the “locked point”. The locked point is then matched to the line defined by the anchor pair and shifted together with its paired point. (12) Two subgroups ({node ➀, node ➁} and {node ➂, node ➃, node ➄, node ➅}) of pairs are formed. (13) Find the point in each subgroup that is closest to the other subgroup. (14) Merge subgroups. (15) Compare the result with the “true” functional form. These steps are being implemented in a semi-automatic algorithm.


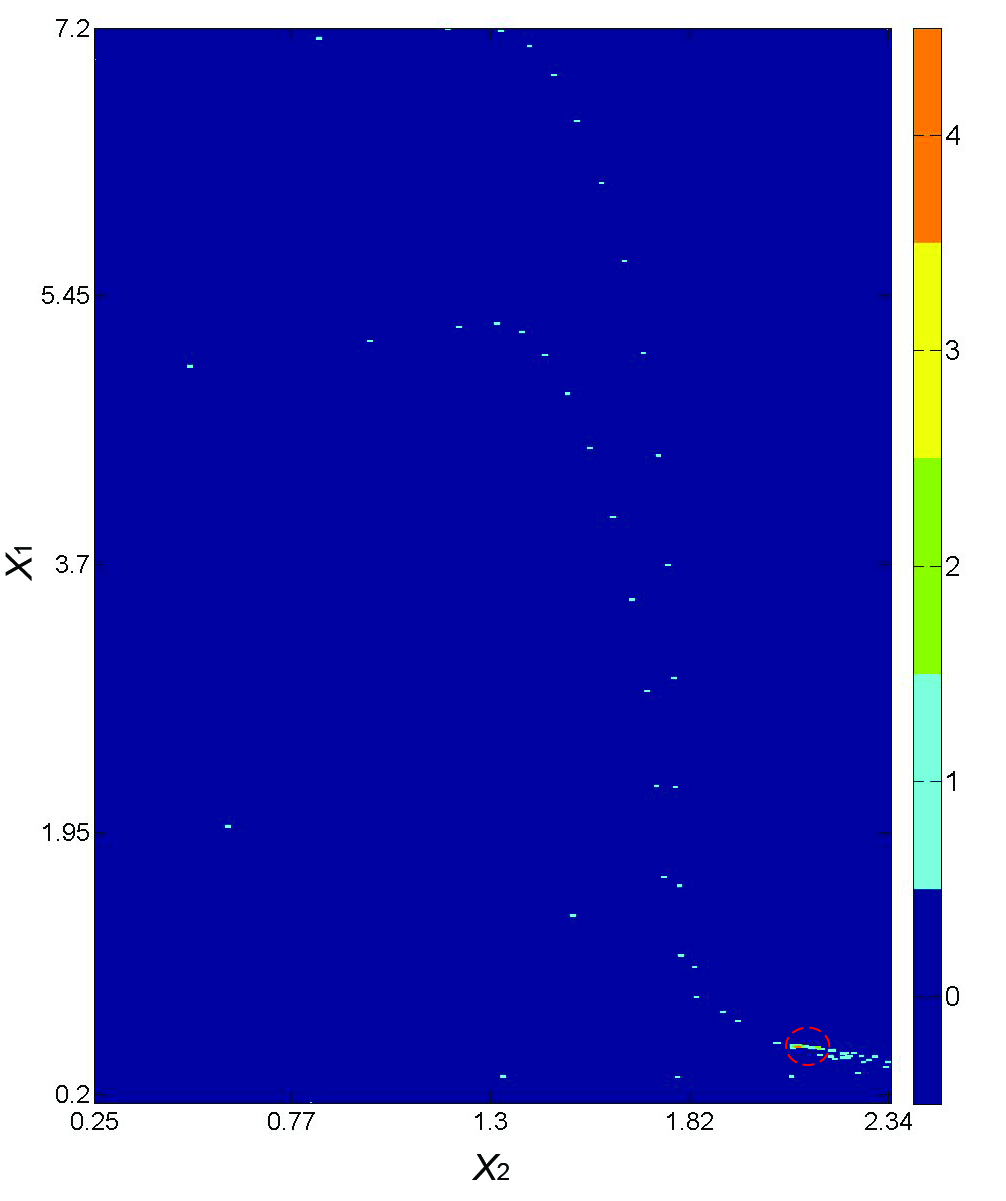


Figure S2. *X*_1_ and *X*_2_ are put into different “grid cells,” based on their values (the size of each grid cell is 0.005×0.005). Here the points are classified into a total of 83 grid cells. Among these, 6 cells have at least two elements (dashed circled area). The color bar shows the number of points in each grid.


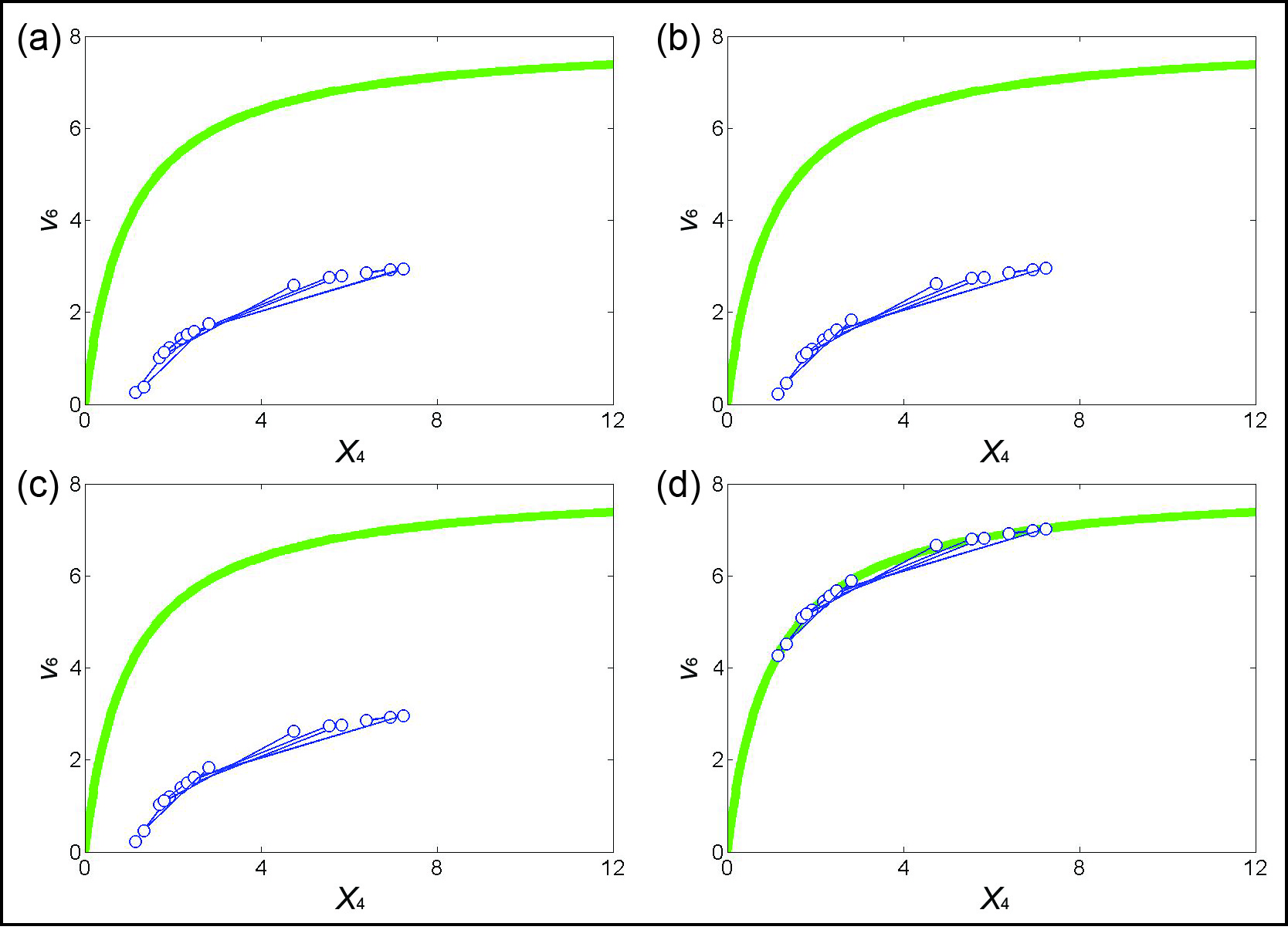


Figure S3. (a) Pieces exceeding threshold *d* (here *s* = 8 and *d* = 0.5; see *Text*). The green line is the “true” measurements of *X*_4_ versus *v*_6_. (b) Merge pairs in (a); the graph becomes connected after this step (see *Methods* for details). (c) Merge subgroups; this figure is the same as (b) since there are no subgraphs; all pairs (nodes) were connected in the previous step. (d) Assume *v*_6_ is known for *X*_4_ $\approx$1.17 and move the entire cluster to match the true measurements of *X*_4_ versus *v*_6_. The sum of errors between the estimated points to their corresponding points in the green line is 0.0199.

Instead of fixing *v*_5_ and solving for *v*_6_, one can solve the fourth equation in the “opposite direction” by fixing *v*_6_. We look for situations within dataset 1 in Fig. 6(b) where *X*_4_ has some fixed value *X*_4c_. Under the assumption of a function, we can assert that *v*_6_ also has some fixed value *v*_6c_. Thus, for the set of all *X*_4_ $\approx$ *X*_4c_ the equation of has the form

. (S2)

Next we screen the dataset for different fixed values of *X*_4_ and put the number of instances into bins based on their *X*_4_ values. For those bins which have at least two points, we find their corresponding *X*_1_ and *X*_2_ and merge the points considering the distances between points and the lengths of pieces in the three-dimensional space. The results are showed in Fig. S4.


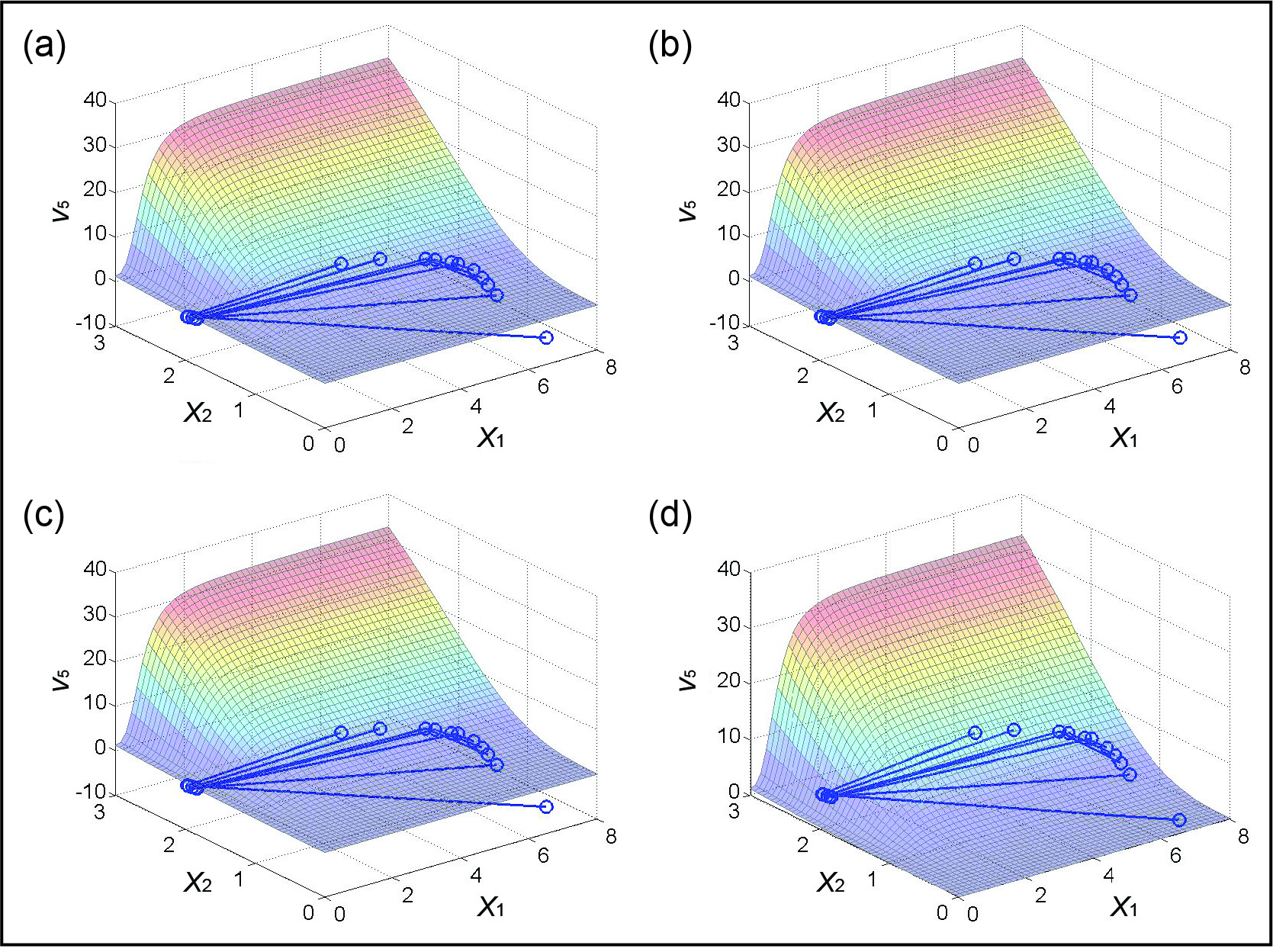


Figure S4. (a) Pieces with *d* > 0.25 (in the direction of *X*_1_) (here *s* = 10). The surface is the “true” but unknown function of *X*_1_ and *X*_2_ versus *v*_5_. (b) Merge pairs in (a). (c) Merge subgroups in (b). (d) Assume *v*_5_ is known at one point of (*X*_1_, *X*_2_); the entire cluster is shifted accordingly to match the true measurements of *X*_4_ versus *v*_6_. The sum of errors between the estimated points and their corresponding points of the surface is 0.2670.

**Estimation from noisy data**

We applied the proposed method to a five-variable system describing an artificial gene network that has been used as a benchmark [[24,31-33](#_ENREF_1)] for S-system inference algorithms (see Eq. (S3)). To test the proposed method for data with noise, we added 5% noise to the artificial time series data. For the illustration we chose the fifth equation in order to determine the *α*-term (*v*_9_) by fixing the *β*-term (*v*_10_) at some values. We randomly selected some datasets from data generated using ten initial conditions (Table S1). The results are shown in Fig. S5.

(S3)

Similarly, we tested the proposed method using datasets which contain different levels of noise. The results are shown in Fig. S6. As to be expected, the quality of the estimated trend is negatively affected by noise. However, one notes that smoothers permit the characterization and amelioration of noise. Thus, effective preprocessing of the data, *i.e.*, noise reduction by means of a filter or smoother (see body of the article), allows us to deal with the issue of noise before applying the method proposed here.

Table S1. Ten sets of initial conditions used in the computational experiments.

| Dataset # | *X*_1_(*t*_0_) | *X*_2_(*t*_0_) | *X*_3_(*t*_0_) | *X*_4_(*t*_0_) | *X*_5_(*t*_0_) |
| --- | --- | --- | --- | --- | --- |
| 1 | 0.70 | 0.12 | 0.14 | 0.16 | 0.18 |
| 2 | 0.10 | 0.70 | 0.14 | 0.16 | 0.18 |
| 3 | 0.10 | 0.12 | 0.70 | 0.16 | 0.18 |
| 4 | 0.10 | 0.12 | 0.14 | 0.70 | 0.18 |
| 5 | 0.10 | 0.12 | 0.14 | 0.16 | 0.70 |
| 6 | 0.70 | 0.70 | 0.14 | 0.16 | 0.70 |
| 7 | 0.10 | 0.70 | 0.70 | 0.16 | 0.18 |
| 8 | 0.10 | 0.12 | 0.70 | 0.70 | 0.18 |
| 9 | 0.10 | 0.12 | 0.14 | 0.70 | 0.70 |
| 10 | 0.70 | 0.12 | 0.14 | 0.16 | 0.70 |


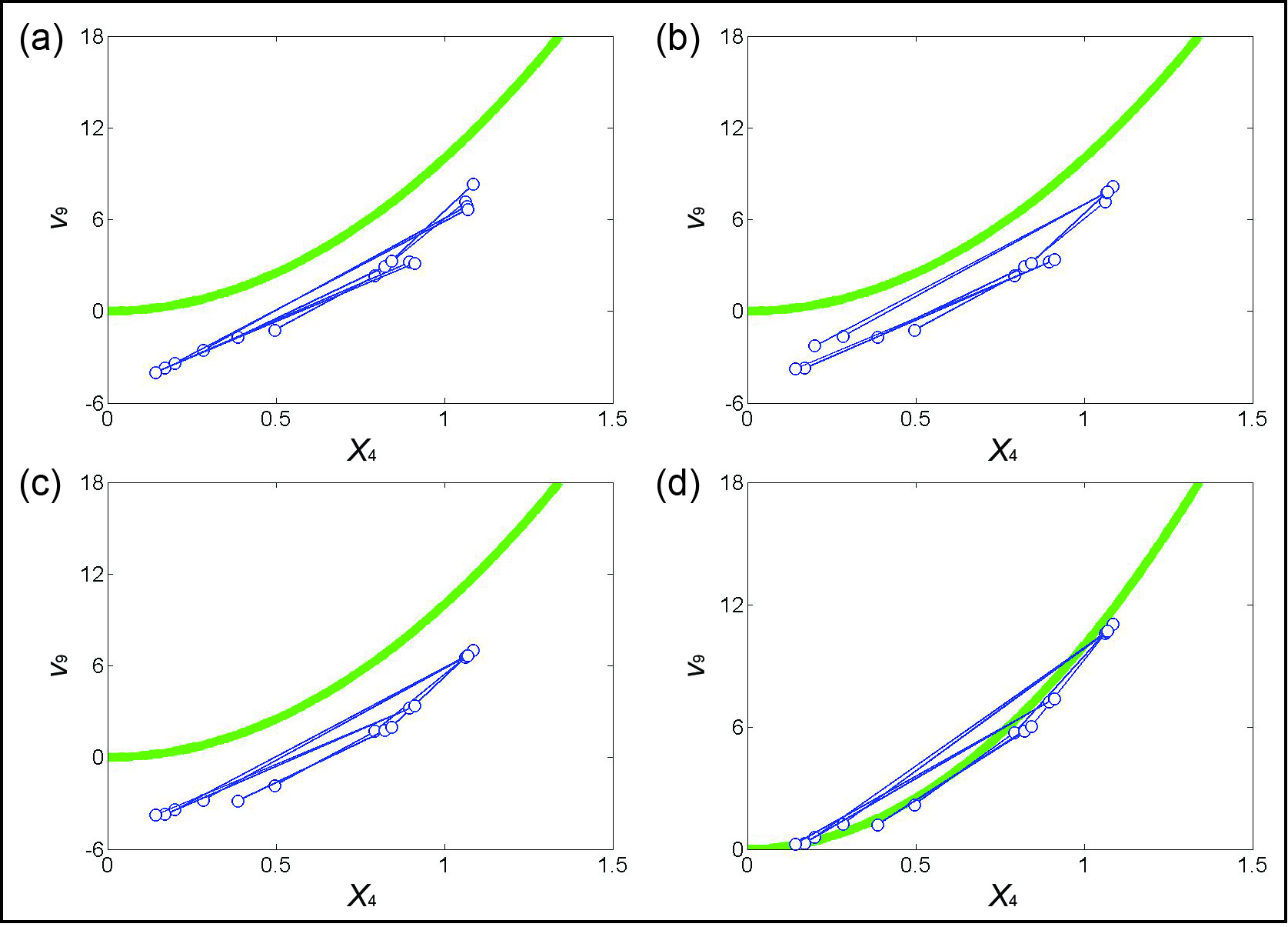


Figure S5. Estimation of the *α*-term (*v*_9_) by fixing the *β*-term (*v*_10_) at some values. Three datasets (#2, #5, and #7) were randomly chosen and used in this example. Each dataset contains 5% random noise in the time series data. (a) Pieces exceeding threshold *d* (here *s* = 8 and *d* = 0.15; see *Text*). The green line is the “true” measurements of *X*_4_ versus *v*_9_. (b) Pairs in (a) are merged, based on the distances between points in each “node” and the distances between two points in a pair. (c) Merge subgroups in (b). (d) Assume *v*_9_ is known for *X*_4_ $\approx$0.15 and move the entire cluster to match the true measurements of *X*_4_ versus *v*_9_.


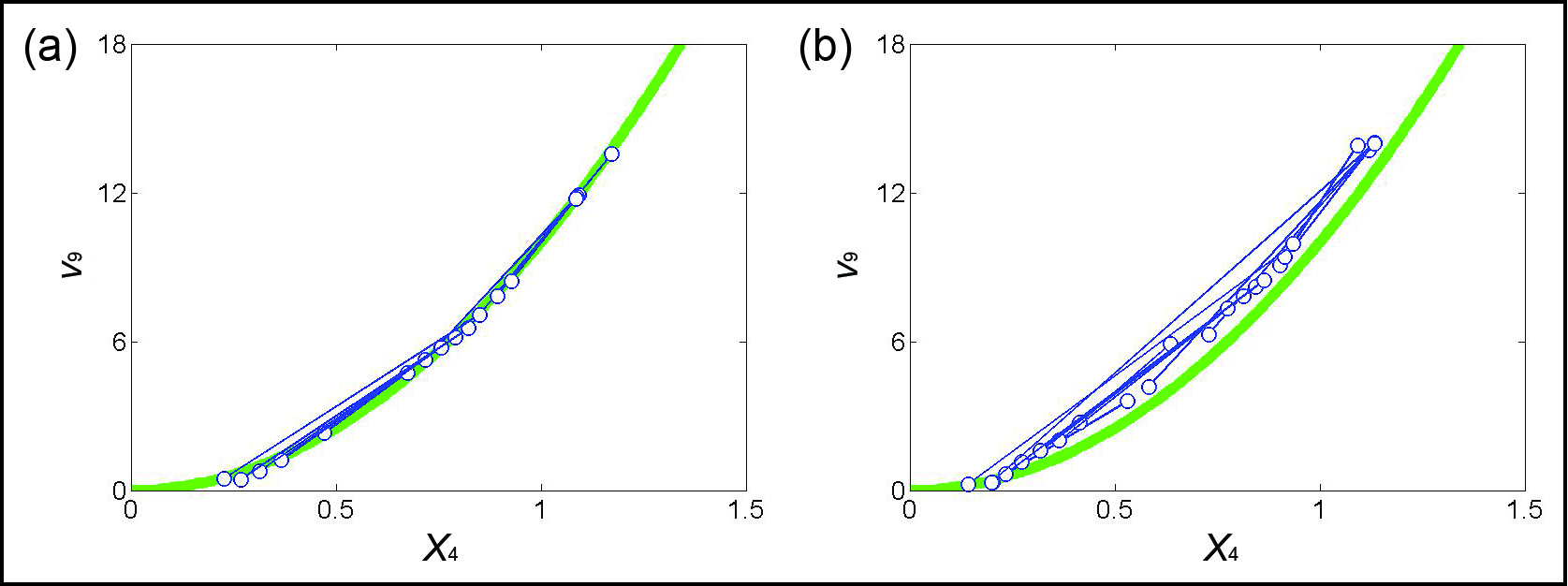


Figure S6. Estimation of the *α*-term (*v*_9_) by fixing the *β*-term (*v*_10_) at some values. The green line is the “true” representation of *X*_4_ versus *v*_9_. (a) Three datasets (#3, #7, and #10) were randomly chosen and used in this example. Each dataset contains 1% of random noise in the time series data. (b) Three datasets (#1, #4, and #10) were randomly chosen and used in this example. Each dataset contains 10% of random noise in the time series data.
